# Learning curve and surgical outcome of robotic assisted colorectal surgery with ERAS program

**DOI:** 10.1038/s41598-022-24665-w

**Published:** 2022-11-29

**Authors:** Chun-Yu Lin, Yi-Chun Liu, Ming-Cheng Chen, Feng-Fan Chiang

**Affiliations:** 1 Department of Colorectal Surgery, Taichung Veterans General Hospital, Taichung, Taiwan; 2 School of Medicine, National Defense Medical University, Taipei, Taiwan; 3 Institute of Clinical Medicine, National Yang Ming Chiao Tung University, Taipei, Taiwan; 4 College of Humanities and Social Sciences, Providence University, Taichung, Taiwan; 5 Department of Radiation Oncology, Taichung Veterans General Hospital, Taichung, Taiwan; 6 School of Medicine, National Yang Ming Chiao Tung University, Taipei, Taiwan; 7 Department of Post-Baccalaureate Medicine, College of Medicine, National Chung Hsing University, Taichung, Taiwan

**Keywords:** Gastrointestinal diseases, Surgical oncology

## Abstract

This study analyzed learning curve and the surgical outcome of robotic assisted colorectal surgery with ERAS program. The study results serve as a reference for future robotic colorectal surgeon who applied ERAS in clinical practice. This was a retrospective case–control study to analyze the learning curve of 141 robotic assisted colorectal surgery (RAS) by Da Vinci Xi (Xi) system and compare the surgical outcomes with 147 conventional laparoscopic (LSC) surgery in the same team. Evaluation for maturation was performed by operation time and the CUSUM plot. Patients were recruited from 1st February 2019 to 9th January 2022; follow-up was conducted at 30 days, and the final follow-up was conducted on 9th February 2022. It both took 31 cases for colon and rectal robotic surgeries to reach the maturation phase. Teamwork maturation was achieved after 60 cases. In the maturation stage, RAS required a longer operation time (mean: colon: 249.5 ± 46.5 vs. 190.3 ± 57.3 p < 0.001; rectum 314.9 ± 59.6 vs. 223.6 ± 63.5 p < 0.001). After propensity score matching, robotic surgery with ERAS program resulted in significant shorter length of hospital stay (mean: colon: 5.5 ± 4.5 vs. 10.0 ± 11.9, p < 0.001; rectum: 5.4 ± 3.5 vs. 10.1 ± 7.0, p < 0.001), lower minor complication rate (colon: 6.0% vs 20.0%, p = 0.074 ; rectum: 11.1% vs 33.3%, p = 0.102), and no significant different major complication rate (colon: 2.0% vs 6.0%, p = 0.617; rectum: 7.4% cs 7.4%, p = 1.0) to conventional LSC. Learning curve for robotic assisted colorectal surgery takes 31 cases. Robotic surgery with ERAS program brings significant faster recovery and fewer complication rate compared to laparoscopy in colorectal surgery.

## Introduction

Robotic surgery was first announced as a tool for remote-controlled surgery in astronauts on spaceship missions. Today, it is widely used in operating rooms, worldwide, because it is an advanced micro-invasive surgery instrument that results in fewer complications than open surgery^1^. The system that was the earliest developed and most used today was the Intuitive Da Vinci robot, which announced its 4th generation Xi system in 2015 and has become tremendously popular in the field of gastrointestinal surgery during these years^2^.

Compared to conventional laparoscopic instruments, robotic systems have advantages in movable endo-wrist systems capable of performing delicate movements. The camera was integrated into the console, which was controlled by the surgeon, thus avoiding uncertainty and inaccuracy. However, due to the longer setup time, limited surgical field, and higher resource expenses, only limited surgeons use the system. In the beginning to do robotic colorectal surgery, surgeons might encounter complicated steps, collision between robotic arms and long operative times, which results in a stressful work environment. Fortunately, the problems are alleviated by using the DaVinci Xi systems. The system improved the range of motion of each arm, thus allowing the arm to be slim and flexible. There were energy devices and endoscopic anastomosis stapler released which help the gastrointestinal surgery more easily.

This study aims to determine the learning curve of the transition from laparoscopic to robotic assisted colorectal surgery using the Xi system, and to analyze the surgical outcomes. We hypothesize that it takes 30 cases to complete the transformation to this platform and that robotic surgery may result in better recovery.

## Materials and methods

This is a retrospective case–control study. To analyze the learning curve of robotic naïve laparoscopy surgeons performing colorectal surgery by using the DaVinci Xi system, and to analyze the surgical outcomes during this period.

Since ^1^ February 2019, the robotic team of the colorectal surgery department in TCVGH led by FFC (A) and another physician CYL (B) have performed robotic colorectal surgery. Surgeon A was a colorectal surgeon with 20 years of experience who completed more than 2000 laparoscopies and 10 robot-assisted surgeries using the Si system. Surgeon B was a surgeon with 5 years of experience who completed over 200 laparoscopies and 1 robot-assisted surgery using the Si system. In the same period, they also performed conventional laparoscopy. The robot surgery procedures were on the base of laparoscopy and were conducted under experienced laparoscopy physicians. The physicians complete initial dry lab and animal lab course for robot surgery. The initial robot surgery was supervised by experienced tutors to ensure the safety and quality for the patients. The perioperative management was guided in regular clinical practice and Enhanced Recovery After Surgery (ERAS) protocol were added since 1st Jan 2020. The procedures were all carefully performed with the intention to treat and to do no harm principles in accordance with Declaration of Helsinki. The medical team acknowledge the robot surgery and the informed consents for surgery were agreed by the patients. The preference to choose robotic surgery or laparoscopy was according to the surgeon’s discretion and the patients’ selection.

The primary outcome of this study was the learning curve for the surgeon being familiar with robot systems. The second outcomes were the surgical outcomes of laparoscopic and robotic assisted surgery. The inclusion criteria for the robot group were colorectal surgery performed with the Xi robotic system by Surgeons A and B. Other robot systems or surgeries by other surgeons were excluded. The inclusion criterion for the laparoscopy group was conventional laparoscopic assisted colorectal surgery. Emergent surgeries, open surgeries, trans-anal total mesorectum excision (taTME) surgeries, recurrent disease surgeries and combined surgeries for non-colorectal diseases were excluded. We collected the medical records, surgery videos, and pathology reports to perform the research. The patients’ characteristics and operation types between robot and laparoscopy were matched for further surgical outcome evaluation.

The study was approved on IRB as title “Robotic assisted colon and rectal surgery prognostic and outcome analysis” with IRB number: TCVGH-IRB CE21319A. Register number on ClinicalTrails.gov was NCT05210647. The learning curve evaluation was based on the total operation time (OT), which was recorded on the operation room bulletin. To minimize the fluctuation of the data, the ten running average method was used to analyze the trend of operation time consumption. CUSUM; Cumulative Sum Control Chart as evaluation method for new manufacturing process. The turning points from the valley on CUSUM indicate maturation of the techniques.

Most videos of robot surgery were also reviewed. The root vessel ligation time started from opening the retroperitoneum and ended at vessel resection. Then, the colectomy time followed root ligation and ended at specimen resection. The composition of operation time (OT) was assumed to be root ligation time, colectomy time, combined surgery time and para-surgery time. Para-surgery time was calculated to evaluate the maturation of teamwork.

$$Rootligation\, + \,Colectomy\, + \,Combine \, surgery\, + \,para - operation \, time\, = \,operation \, time \, \left( {OT} \right).$$

The surgical outcome data included total operation time (OT), estimated blood loss (EBL), conversion to open rate (Open), length hospital stay (LHS), and minor and major complication rates. To obtain homogeneity, the research subjects were divided into colon surgery and rectal surgery. The complications were classified as Calvin-Dindo methods. Grade 1–2 was minor complications that took medications. Antiemetics, analgesia, and antidiarrhea were allowed in grade 1. Bedside wound dressing changes and Foley catheter placement were also grade 1. Other events were classified grade 2. Grade 3–4 were major complications that required radiological or surgical interventions under anesthesia. If decompensation of organ function appeared and the patients were admitted to the ICU, their classification was grade 4. Grade 5 was postoperative mortality. The margins, including circumferential margins and distal margins, were also recorded.

According to previous research, it takes 20–40 cases for surgeons to familiarize themselves with robot surgery. We collected approximately 70 colon and rectal cases, including 30 cases after the learning curve, to observe differences before and after the learning curve.

### Statistical analysis

Quantitative data are expressed as the mean ± SD. One-way analysis of variance (ANOVA) with least significant difference (LSD) multiple comparison was used to analyze the quantitative differences between two groups. Groups were compared using t tests, Mann–Whitney U tests, Chi-square tests, and Fisher’s exact tests as appropriate. Statistical analyses were performed with IBM SPSS 20.0 software (SPSS Inc., Chicago, IL, USA). Significance was set at p < 0.05. Propensity score matching (PSM) matched gender, age, ASA score, lesion sideness and neoadjuvant radiotherapy.

## Results

**Figure 1:**
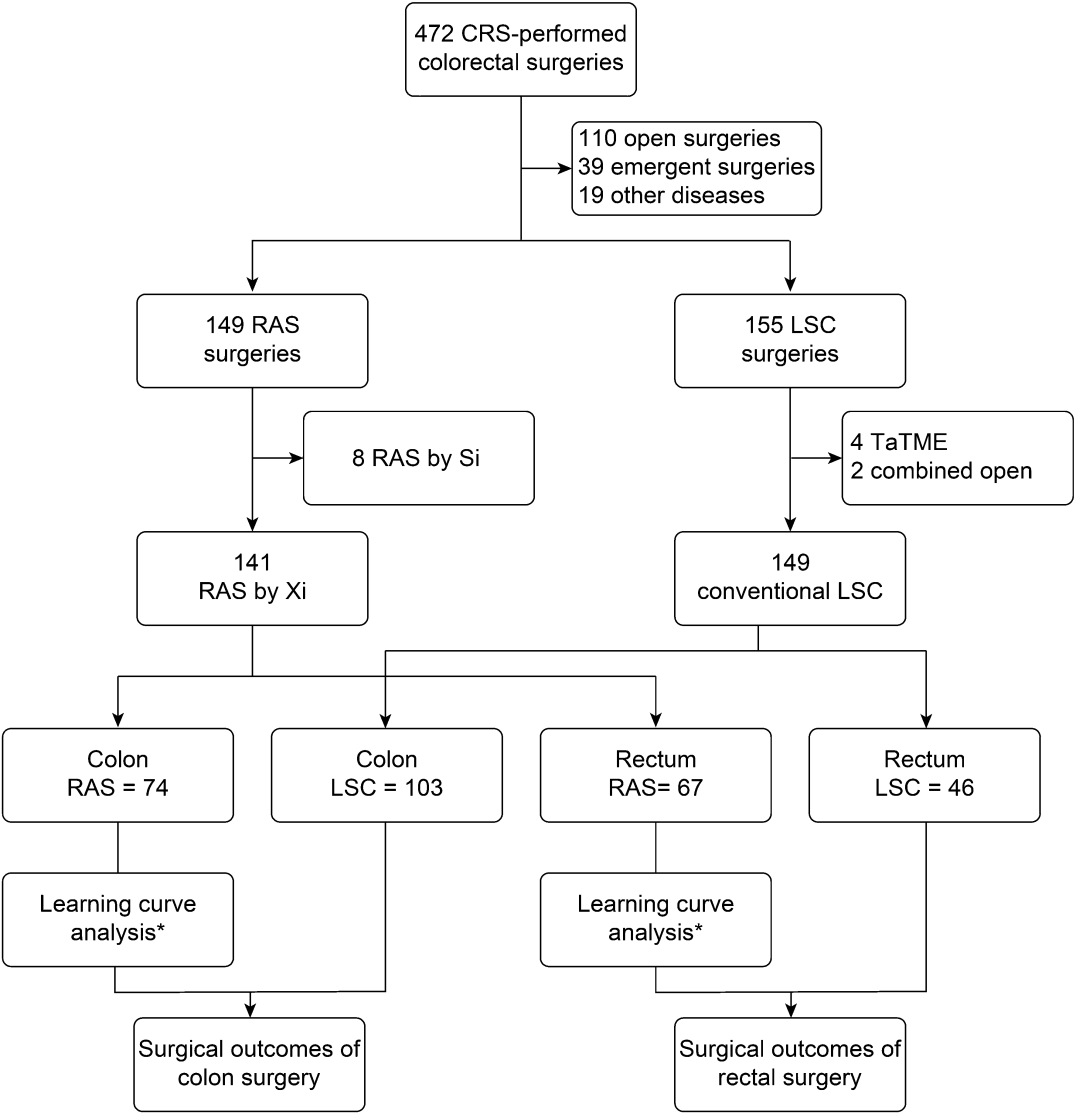
Schematic illustration for study materials and methods. Number of cases from each kind of surgery. The flow of included cases and the study methods. *CRS* colorectal surgery, *RAS* robotic assisted surgery, *LSC* laparoscopic surgery, *Si* da Vinci Si, *Xi* da Vinci Xi, *TaTME* trans-anal total mesorectum excision.

From 1 February 2019 to 9 January 2022, there were 472 colorectal surgeries performed by the surgical team. A total of 110 open surgeries, 39 emergency operations, 4 taTME surgeries, and 19 other surgeries with origin diseases were excluded in the initial steps. There were 149 RAS colorectal surgery and 155 LCS colorectal surgeries. Then, after excluding 8 cases by the da Vinci Si (Si) system in the robot group and 4 taTME surgeries, 2 laparoscopic surgeries combined with open hepatectomy were performed in the laparoscopy arm. There were 141 cases performed by Xi robotic system and 149 conventional LSC colorectal surgeries involved in the study. Lesion sites in the colon and rectum were 74:67 in RAS and 103:46 in LSC. This distribution might be because of the recommendation from physicians to use robots for rectal lesions. Most videos (80.1%, 114/141) of RAS were also reviewed to depict procedure details during the learning curve **(**Fig. 1).

**Figure 2:**
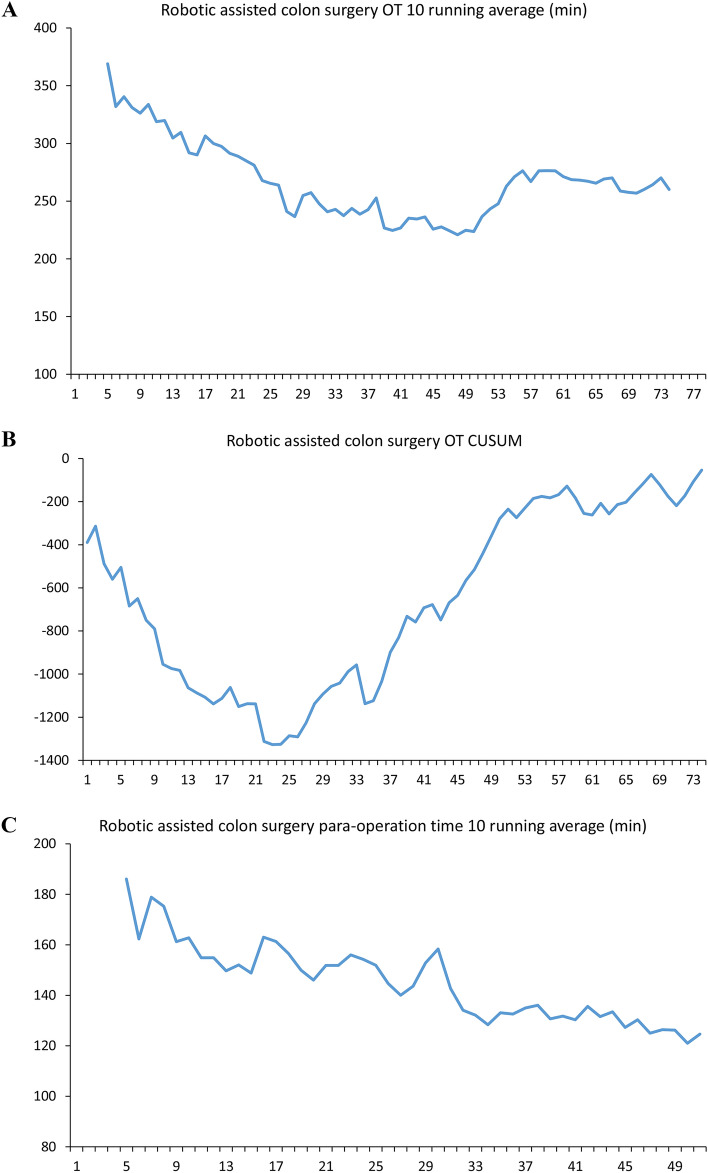
Learning curve analysis of robotic assisted colon surgery. (**A**) Robotic assisted colon surgery operation time 10 running average (min). (**B**) Robotic assisted colon surgery operation time CUSUM plot. (**C**) Robotic assisted colon surgery para-operation time 10 running average (min). *OT* operation time, *CUSUM* cumulative sum control chart.

In robotic colon surgery, the medical team took 31 cases to decrease the operation time to a consistent operation time. The number was compatible with the turning point of the CUSUM slope. The para-surgical time continuously decreased until case number 34 (case 57). This means the maturation of teamwork (Fig. 2).

**Figure 3:**
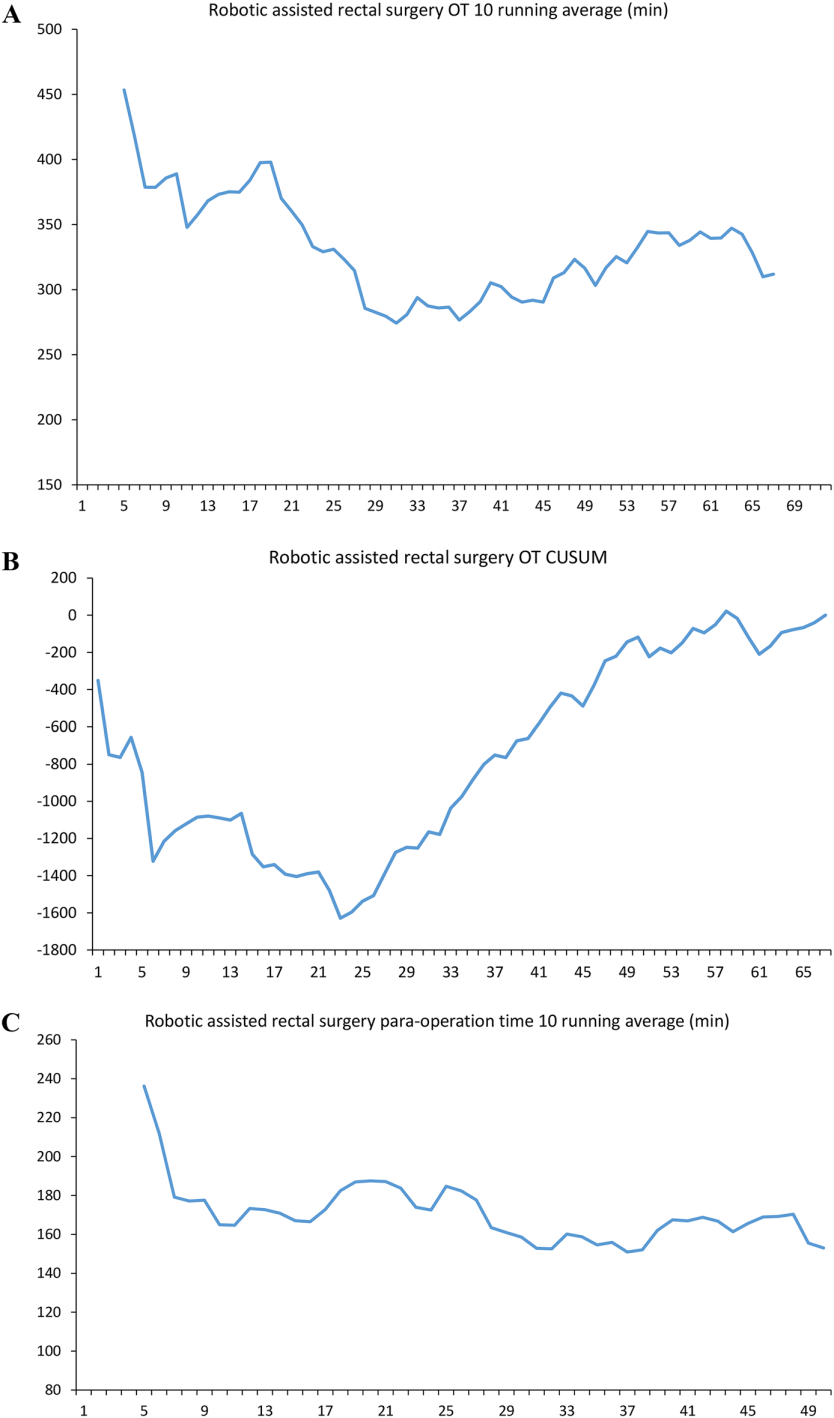
Learning curve analysis of robotic assisted rectum surgery. (**A**) Robotic assisted rectal surgery operation time 10 running average (min). (**B**) Robotic assisted rectal surgery operation time CUSUM plot. (**C**) Robotic assisted rectal surgery para-operation time 10 running average (min). *OT* operation time, *CUSUM* cumulative sum control chart.

In robotic rectal surgery, the team also took 31 cases to decrease the operation time. The number was compatible with the turning point of the CUSUM slope. The para-surgical time decreased until case number 28 (case 67). The maturation of teamwork happens after case 60 (Fig. 3).

For properties of the robotic colon surgery cases (n = 74), there were fewer right-sided lesions (28.4% vs. 39.8%, p = 0.230) compared to the LSC group (n = 103). ERAS protocols were initiated in the institute during this period and were accepted in most robotic surgery patients (82.4% vs. 20.4%, p < 0.001). The study compared LSC and RAS after PSM (age, sex, ASA score and lesion sideness), and also subgroup robotic ERAS and conventional LSC to evaluate the effect of this practice.

For outcomes of robotic colon surgery, the mean operation time (min) was higher than that of LSC (all: 273.9 ± 82.7 vs. 190.3 ± 57.3, p < 0.001; maturation stage: 249.5 ± 46.5 vs. 190.3 ± 57.3, p < 0.001). Mean blood loss (ml) was higher than LSC (all: 88.3 ± 166.8 vs. 49.3 ± 67.8, p = 0.025; maturation stage: 95.4 ± 213.2 vs. 49.3 ± 67.8, p = 0.430) (Supplement Table). After PSM, the length of hospital stay (LHS) was shorter in the RAS group (mean: 6.3 ± 4.6 vs. 9.3 ± 10.2, median: 5.0 (4.0–7.0) vs. 7.0 (5.0–10.0), p < 0.001). The minor complication rate was significantly lower (9.9% vs. 28.2% p = 0.010). The major complication rate was the same (2.8% vs. 2.8%, p = 1.0) (Table 1).

**Table 1:** Demographics and surgical outcomes of LSC and RAS colon surgery. Abbreviations: LSC: laparoscopy; RAS: robotic assisted surgery; PSM: propensity score matching; ERAS: enhanced recovery after surgery; RH: right hemicolectomy; LH: left hemicolectomy; AR: anterior resection.

Colon surgery	Original data				PSM (Gender, age, ASA, lesion sideness)					Robotic ERAS PSM				
	LSC all (n = 103)	RAS all (n = 74)		*p*value	LSC (n = 71)		RAS (n = 71)		*p *value	Convention LSC (n = 50)		Robotic ERAS (n = 50)		*p *value
**Gender**				0.429					1.000					0.838
Male	40 (39%)	34 (46%)			32 (45%)		32 (45%)			19 (38%)		21 (42%)		
Female	63 (61%)	40 (54%)			39 (55%)		39 (55%)			31 (62%)		29 (58%)		
Age	66.0 (53.0–76.0)	62.0 (50.0–72.3)		0.392	66.0 (55.0–75.0)		62.0 (50.0–72.0)		0.176	64.0 (55.8–78.0)		61.5 (50.8–71.3)		0.191
**ASA**				0.827					0.852					0.378
1 + 2	71 (69%)	53 (72%)			50 (70%)		52 (73%)			33 (66%)		38 (76%)		
3	32 (31%)	21 (28%)			21 (30%)		19 (27%)			17 (34%)		12 (24%)		
**BMI**				0.676					0.384					0.841
< 24.9	65 (64%)	44 (59%)			48 (68%)		42 (59%)			26 (52%)		28 (56%)		
≥ 25	37 (36%)	30 (41%)			23 (32%)		29 (41%)			24 (48%)		22 (44%)		
**Disease**				0.677					1.000					0.758
Benign	16 (16%)	9 (12%)			9 (13%)		9 (13%)			7 (14%)		5 (10%)		
Malignant	87 (84%)	65 (88%)			62 (87%)		62 (87%)			43 (86%)		45 (90%)		
Adenocarcinoma	86 (83%)	65 (88%)		0.555	62 (87%)		62 (87%)		1.000	42 (84%)		45 (90%)		0.552
**Lesion sideness**				0.158					1.000					0.809
Right (A, T)	41 (40%)	21 (28%)			20 (28%)		20 (28%)			10 (20%)		12 (24%)		
Left (D, S)	62 (60%)	53 (72%)			51 (72%)		51 (72%)			40 (80%)		38 (76%)		
**Surgery type**				0.405					0.738					0.598
RH	37 (36%)	18 (24%)			19 (27%)		17 (24%)			9 (18%)		11 (22%)		
LH	5 (5%)	4 (5%)			5 (7%)		4 (6%)			4 (8%)		2 (4%)		
AR	59 (57%)	51 (69%)			47 (66%)		49 (69%)			37 (74%)		36 (72%)		
Subtotal	2 (2%)	1 (1%)			0 (0%)		1 (1%)			0 (0%)		1 (2%)		
Combine surgery	16 (16%)	6 (8%)		0.213	12 (17%)		6 (8%)		0.207	5 (10%)		2 (4%)		0.436
**Surgeon**				0.305					0.856					0.127
A	78 (76%)	50 (68%)			50 (70%)		48 (68%)			31 (62%)		39 (78%)		
B	25 (24%)	24 (32%)			21 (30%)		23 (32%)			19 (38%)		11 (22%)		
ERAS	21 (20%)	61 (82%)		< 0.001**	14 (20%)		58 (82%)		< 0.001**	0 (0%)		50 (100%)		< 0.001**
**Operation time**														
Mean	190.3 ± 57	273.9 ± 83		< 0.001**	188.2 ± 58		272.7 ± 82		< 0.001**	186.0 ± 55		257.4 ± 61		< 0.001**
Median	177.0 (150.0–219.0)	259.0 (223.0–306.5)			175.0 (150.0–213.0)		259.0 (220.0–304.0)			175.0 (143.8–216.3)		239.0 (219.8–285.8)		
Stoma+	2 (2%)	3 (4%)		0.403	1 (1%)		3 (4%)		0.366	1 (2%)		1 (2%)		1.000
**Blood loss**														
Mean	49.3 ± 68	88.3 ± 167		0.025*	51.1 ± 65		90.7 ± 170		0.084	39.4 ± 30		74.6 ± 177		0.371
Median	30.0 (30.0–30.0)	30.0 (30.0–35.0)			30.0 (30.0–30.0)		30.0 (30.0–50.0)			30.0 (30.0–30.0)		30.0 (30.0–30.0)		
Open rate	5 (5%)	1 (1%)		0.403	2 (3%)		1 (1%)		1.000	1 (2%)		0 (0%)		1.000
**p stage**				0.119					0.202					0.051
0, 1	26 (32%)	11 (17%)			17 (30%)		10 (16%)			17 (41%)		8 (18%)		
2, 3	48 (59%)	47 (72%)			35 (61%)		46 (74%)			22 (54%)		33 (73%)		
4	8 (10%)	7 (11%)			5 (9%)		6 (10%)			2 (5%)		4 (9%)		
Distal margin, mean	6.1 ± 5.7	4.3 ± 2.7		0.054	5.4 ± 4.4		4.3 ± 2.7		0.166	4.8 ± 3.8		4.4 ± 2.6		0.955
**Hospital stay**														
Mean	9.2 ± 9.6	6.4 ± 4.8		< 0.001**	9.3 ± 10.2		6.3 ± 4.6		< 0.001**	10.0 ± 11.9		5.5 ± 4.5		< 0.001**
Median	6.0 (5.0–10.0)	5.0 (4.0–7.0)			7.0 (5.0–10.0)		5.0 (4.0–7.0)			7.0 (6.0–9.3)		4.0 (4.0–6.0)		
**Complication**														
Minor	23 (22%)	7 (9%)		0.041*	20 (28%)		7 (10%)		0.010*	10 (20%)		3 (6%)		0.074
Major	4 (4%)	2 (3%)		1.000	2 (3%)		2 (3%)		1.000	3 (6%)		1 (2%)		0.617
Leakage rate	1 (1%)	3 (4%)		0.310	1 (1%)		3 (4%)		0.620	1 (2%)		2 (4%)		1.000

Chi-square test or Mann–Whitney U test.

*p < 0.05, **p < 0.01.

For properties of the robotic rectal surgery cases (n = 67), younger age (56.0 vs. 65.0, p < 0.001), fewer ASA 3 patients (19.4% vs. 37.0%, p = 0.063), more local advanced lesions (37.5% vs. 28.6%, p = 0.112), more were at low rectal site (37.5% vs. 28.6%, p = 0.459), significant more post radiotherapy (50.0% vs. 21.4%, p = 0.006) and more ERAS protocol (80.6% vs. 8.7%, p < 0.001) (Table 2).

**Table 2:** Demographics and surgical outcomes of LSC and RAS rectal surgery. Abbreviations: LSC: laparoscopy; RAS: robotic assisted surgery; PSM: propensity score matching; ERAS: enhanced recovery after surgery; U/3: upper third; M/3: middle third; L/3: low third; RT: radiotherapy; LAR: low anterior resection; TME: total mesorectal excision; APR: abdominoperineal resection; CRM: circumferential margin.

Rectal surgery	Original			PSM (gender, age, ASA, radiotherapy)			Robotic ERAS PSM		
	LSC all (n = 46)	RAS all (n = 67)	*p*value	LSC (n = 32 )	RAS (n = 32 )	*p*value	Convention LSC (n = 27)	Robotic ERAS (n = 27)	*p*value
**Gender**			0.635			0.796			0.406
Female	15 (33%)	26 (39%)		11 (34%)	13 (41%)		9 (33%)	13 (48%)	
Male	31 (67%)	41 (61%)		21 (66%)	19 (59%)		18 (67%)	14 (52%)	
Age	65.0 (61.0–73.8)	56.0 (47.0–68.0)	< 0.001**	63.0 (59.5–70.5)	65.5 (54.5–74.0)	0.824	64.0 (62.0–71.0)	67.0 (54.0–71.0)	0.945
**ASA**			0.063			1.000			1.000
1 + 2	29 (63%)	54 (81%)		24 (75%)	24 (75%)		19 (70%)	20 (74%)	
3	17 (37%)	13 (19%)		8 (25%)	8 (25%)		8 (30%)	7 (26%)	
**BMI**			1.000			0.497			0.904
< 24.9	28 (62%)	42 (64%)		18 (56%)	21 (68%)		16 (59%)	14 (54%)	
≥ 25	17 (38%)	24 (36%)		14 (44%)	10 (32%)		11 (41%)	12 (46%)	
**Disease**			0.714			1.000			1.000
Benign	4 (9%)	4 (6%)		0 (0%)	1 (3%)		0 (0%)	1 (4%)	
Malignant	42 (91%)	63 (94%)		32 (100%)	31 (97%)		27 (100%)	26 (96%)	
cT3 margin+	12 (29%)	24 (38%)	0.459	11 (34%)	12 (38%)	1.000	7 (26%)	6 (22%)	1.000
**Lesion site**			0.448			0.608			0.782
U/3	17 (37%)	19 (28%)		11 (34%)	14 (44%)		10 (37%)	12 (44%)	
M/3 + L/3	29 (63%)	48 (72%)		21 (66%)	18 (56%)		17 (63%)	15 (56%)	
**Pre-op RT**			0.006**			1.000			0.524
No	33 (79%)	32 (50%)		23 (72%)	23 (72%)		19 (70%)	22 (81%)	
Yes	9 (21%)	32 (50%)		9 (28%)	9 (28%)		8 (30%)	5 (19%)	
**Intention surgery**			0.914			0.695			0.757
LAR	15 (33%)	20 (30%)		9 (29%)	12 (38%)		10 (37%)	12 (44%)	
TME	24 (53%)	40 (60%)		16 (52%)	17 (53%)		13 (48%)	13 (48%)	
APR	4 (9%)	5 (7%)		4 (13%)	2 (6%)		3 (11%)	1 (4%)	
Others	2 (4%)	2 (3%)		2 (6%)	1 (3%)		1 (4%)	1 (4%)	
Combine surgery	6 (13%)	6 (9%)	0.543	4 (13%)	3 (9%)	1.000	5 (19%)	2 (7%)	0.420
**Surgeon**			0.339			0.708			0.669
A	41 (89%)	54 (81%)		29 (91%)	27 (84%)		23 (85%)	25 (93%)	
B	5 (11%)	13 (19%)		3 (9%)	5 (16%)		4 (15%)	2 (7%)	
ERAS	4 (9%)	54 (81%)	< 0.001**	3 (9%)	25 (78%)	< 0.001**	0 (0%)	27 (100%)	< 0.001**
**Operation time**									
Mean	223.6 ± 64	345.3 ± 115	< 0.001**	225.9 ± 66	339.9 ± 108	< 0.001**	228.2 ± 66	317.8 ± 73	< 0.001**
Median	210.0 (175.0–270.0)	319.0 (273.0–360.0)		210.0 (175.0–273.0)	319.0 (264.5–366.7)		210.0 (175.0–274.0)	329.0 (259.0–360.0)	
Stoma+	22 (48%)	52 (79%)	0.001**	17 (53%)	23 (72%)	0.197	14 (52%)	18 (69%)	0.311
**Blood loss**									
Mean	78.3 ± 77	82.7 ± 135	0.428	89.1 ± 86	62.8 ± 57	0.349	77.4 ± 75	44.1 ± 41	0.083
Median	30.0 (30.0–112.5)	30.0 (30.0–100.0)		30.0 (30.0–150.0)	30.0 (30.0–100.0)		30.0 (30.0–150.0)	30.0 (30.0–30.0)	
Open rate	3 (7%)	0 (0%)	0.065	3 (9%)	0 (0%)	0.238	1 (4%)	0 (0%)	1.000
**p stage**			0.689			0.690			0.558
0, 1	15 (36%)	28 (43%)		13 (41%)	11 (34%)		11 (41%)	13 (48%)	
2, 3	24 (57%)	34 (52%)		17 (53%)	20 (63%)		13 (48%)	13 (48%)	
4	3 (7%)	3 (5%)		2 (6%)	1 (3%)		3 (11%)	1 (4%)	
CRM positive	2 (4%)	7 (10%)	0.310	2 (6%)	4 (13%)	0.672	2 (7%)	2 (7%)	1.000
Distal margin involved	3 (7%)	0 (0%)	0.065	2 (6%)	0 (0%)	0.492	2 (7%)	0 (0%)	0.491
Distal margin, mean	2.2 ± 1.7	2.2 ± 2.1	0.965	2.1 ± 1.7	2.3 ± 1.9	0.585	2.2 ± 2.0	2.1 ± 1.4	0.676
**Hospital stay**									
Mean	10.9 ± 7.4	6.8 ± 5.7	< 0.001**	9.1 ± 6.4	6.3 ± 4.1	< 0.001**	10.1 ± 7.0	5.4 ± 3.5	< 0.001**
Median	8.0 (6.0–13.3)	5.0 (4.0–7.0)		7.0 (6.0–8.0)	5.0 (4.0–7.0)		7.0 (6.0–11.0)	4.0 (4.0–5.0)	
**Complication**									
Minor	17 (37%)	8 (12%)	0.004**	9 (28%)	4 (13%)	0.214	9 (33%)	3 (11%)	0.102
Major	3 (7%)	7 (10%)	0.524	2 (6%)	3 (9%)	1.000	2 (7%)	2 (7%)	1.000
Leakage rate	1 (2%)	5 (7%)	0.398	0 (0%)	2 (6%)	0.492	0 (0%)	2 (7%)	0.491

Chi-square test or Mann–Whitney U test.

*p < 0.05, **p < 0.01.

For outcomes of robotic rectal surgery, the mean operation time was longer (all: 345.3 ± 115.1 vs. 232.6 ± 63.5; maturation stage 314.9 ± 59.6 vs. 223.6 ± 63.5). Mean blood loss (ml) was similar and tended to be lower in RAS (all: 82.7 ± 135.1 vs. 78.3 ± 76.8, p = 0.428; maturation stage: 46.5 ± 34.8 vs. 78.3 ± 76.8, p = 0.127) (Supplement Table). After PSM (age, sex, ASA score, preoperative RT), there was no difference in stoma creation (71.9% vs. 53.1%, p = 0.197), open rate (0.0% vs. 9.4%, p = 0.238), CRM positive rate (12.5% vs. 6.3%, p = 0.672) or distal margin involvement rate (0% vs. 6.3%, p = 0.492). A higher proportion of intense neoadjuvant therapy like concurrent chemotherapy (CCRT) and total neoadjuvant therapy (TNT) patients represent more severity of disease in RAS rectal surgery which influencing CRM positive rate. LHS was significantly shorter in RAS (mean: 6.3 ± 4.1 vs. 9.1 ± 6.4, median: 5.0 (4.0–7.0) vs. 7.0 (6.0–8.0), p < 0.001). The minor complication rate was lower in RAS (all: 11.9% vs. 37.0% p = 0.004; PSM: 12.5% vs. 28.1%, p = 0.214). The major complication rate was not different (all: 10.4% vs. 6.5%, p = 0.524, PSM: 9.4% vs. 6.3%, p = 1.000). The leakage rate was not significant in the PSM groups (6.3% vs. 0.0%, p = 0.492) (Table 2).

For robot patients with ERAS program, length of hospital stay was significant shorter in both colon (mean: 5.5 ± 4.5 vs. 10.0 ± 11.9, median: 4.0 (4.0–6.0) vs. 7.0 (6.0–9.3), p < 0.001) and rectal (mean: 5.4 ± 3.5 vs. 10.1 ± 7.0, median: 4.0 (4.0–5.0) vs. 7.0 (6.0–11.0), p < 0.001) surgery to conventional LSC. Minor complication rate was lower without significant in robotic ERAS for colon (6% vs 20.0%, p = 0.076) and rectal (11.1% vs 33.3%, p = 0.102) surgery. Major complication rate was no significant difference in colon (2.0% vs 6.0%, p = 0.617) and rectal (7.4% cs 7.4%, p = 1.0) surgery.

## Discussion

In this study, an experienced laparoscopy colorectal surgeon using the Da Vinci Xi began the robotic surgery which is superior in range of motion and is capable of completing multiquadrant surgery^3^. Protyniak et al.^4^ reported using Xi to complete spleen flexure mobilization during low anterior resection in single docking. There were attempts to perform spleen mobilization in single docking in 4 cases. There were 2 cases of failure. The low rectal lesions forced the console to target deep into the pelvis and limited its possibility to achieve spleen flexure. In addition, the Trendelenburg position for pelvis surgery also causes upward colon in the spleen flexure area. This procedure is possible, but its effect is not guaranteed. With the integrated table motion (ITM) system, a good candidate can increase the success rate.

**Figure 4:**
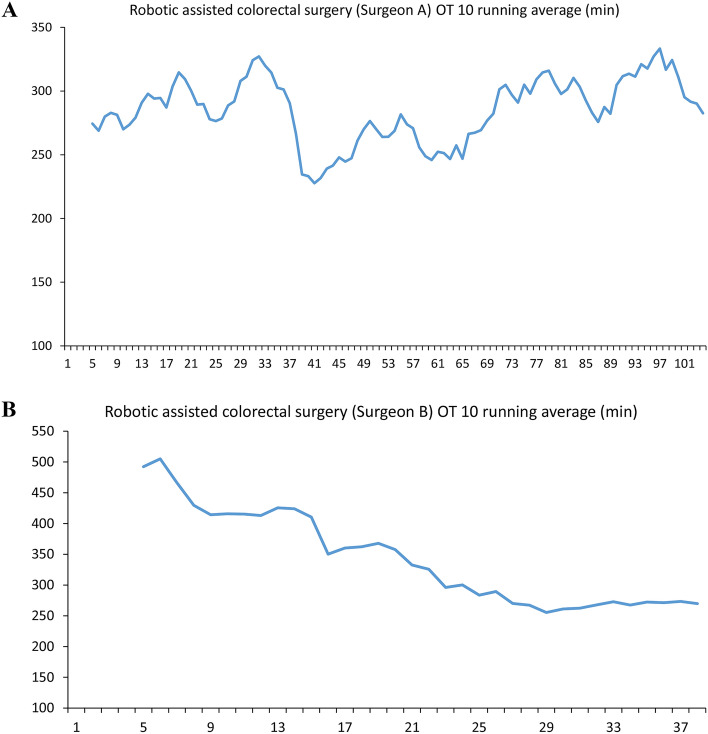
Personal learning curve for robotic assisted colorectal surgery. Personal learning curve for robotic assisted colorectal surgery by da Vinci Xi^®^. Robotic assisted colorectal surgery operation time 10 running average (min) by Surgeon A (FFC: 20 years experienced complete 2000 laparoscopy surgery). Robotic assisted colorectal surgery operation time 10 running average (min) by Surgeon B (CYL: 5 years experienced complete 200 laparoscopy surgery).

There are three stages of the learning curve for robotic surgeons: 1–15 beginning stage, 16–25 stabilizing stage, and after 25 maturing stage^5^. In this study, maturation both took 30 cases to for colon and rectal surgery. This number was similar to that in a previous study using Si system^6–8^. The case number needed to achieve the mature stage at this site was 60, which is also similar to a previous study^9,10^. Maturation was earlier in robotic colon surgery in whole RAS cases (57 vs 67) which is comparable to the recent study^11^ showed faster competent stage for sigmoid colectomy (n = 25) than low anterior resection (n = 41). Numerous cases and operating times were needed to become familiar with RAS. However, there was a very low incidence of machinery problems. For the personal learning curve of each surgeon, well-experienced and young laparoscopy surgeons achieved their mature stage during 25–40 cases (Fig. 4). Previous laparoscopy experience did not affect robot surgery maturation, which is also correlated with study^12^.

Choosing the case is important for every surgeon beginning robotic surgery. It is challenging to start a new surgery or use new equipment. The pioneer gaining skillful knowledge must also establish teamwork. The following member can be easier on the previous basis. Bencini et al.^13^ suggested beginning robotic right hemicolectomy (RRH) to reduce the learning curve for more challenging procedures, such as robotic rectal resection (RRR). However, the development of robot systems in simple surgery regarding the standard laparoscopic approach is less evident compared to complex cases^14^. Surgeon A performed standard surgery in the initial step, and surgeon B chose complex surgery in the initial step. Although the maturation case numbers were 40 and 25, the total operation times were 191.12 h and 177.15 h, respectively. Perhaps neither the case number nor the time consumption can faithfully represent the details of the maturation process. When the surgeon feels competent after the initial phase, they are more likely to try more complex surgery, which takes a longer time and has a higher complication risk. This is another process of maturation that goes to the mastering stage. In this study, several advanced laparoscopic techniques were attempted under a robotic system. For example, complete mesocolon excision in RRH, intracorporal anastomosis (ICA), pelvic lymph node dissection, and para-aortic lymph node dissection. These procedures were found to be easier than laparoscopy.

Regarding surgical outcomes, the operation time takes longer for robotic surgery. This result is like those of other studies^15^. Using Xi takes a similar operation time to Si^4^. Although some studies showed similar operation times between robot and laparoscopy^16^, it was not shown at the end of the study. In the maturation stage, the robot system takes 90 min longer for rectal surgery (314.9 ± 59.6 vs. 223.6 ± 63.5) and 60 min longer for colon surgery (249.5 ± 46.5 vs. 190.3 ± 57.3).

Studies about the conversion to the open rate were unable to show significance^15^. There were many confounding factors, and the subjects required a large case number to achieve sample power. Zhang et al.^17^ found robots in colorectal surgery (n = 1466) with a lower open rate in a meta-analysis study. Zhu et al.^18^ also found robot colorectal surgery (n = 472) with a lower open rate in the American College of Surgeons National Surgical Quality Improvement Program database. In this study, the open rate was lower without significance for both colon surgery (all: 1.4% vs. 4.9%, p = 0.403) and rectal surgery (all: 0% vs. 6.5%, p = 0.065). The endo-wrist of the robot system capable of performing adhesion lysis better contributed to reducing the open rate. There was one case of a lysis procedure for bowel obstruction due to an adhesion band, and 23 (16.31%) cases noted moderate adhesion during the surgery. All of them were successfully completed.

Many robotic studies have shown the superiority of LHS. Meta-analysis^17^ found significantly lower LHS (SMD = −0.18, 95% CI −0.32 to −0.05) compared to that in the LSC group. However, in the RCT study, the ROLLAR trial^15^ showed a similar LHS between RAS and LSC rectal surgery (mean 8.0 vs. 8.2). In this study, after PSM, a shorter LHS was found in the RAL group both in colon (6.3 ± 4.6 vs. 9.3 ± 10.2 p < 0.001) and rectal (6.3 ± 4.1 vs. 9.1 ± 6.4, p < 0.001) surgery. The benefits surpassed in robotic ERAS compared to conventional LSC (colon: 5.5 ± 4.5 vs. 10.0 ± 11.9, p < 0.001; rectum: 5.4 ± 3.5 vs. 10.1 ± 7.0, p < 0.001). The steady platform of the robotic system enables the reduction of perioperative injury and postoperative pain, which is attributed to a shorter LHS. In the previous ERAS study^19,20^, there was a 2.5-day shorter LHS in open surgery and a 1.0-day shorter LHS in laparoscopy surgery (5 vs. 6). In this study, we can conclude that robotic surgery with ERAS program brings 4.5-day significant faster recovery than conventional LSC.

The comparison of complications between RAS and LSC was mostly similar in the past. The overall rates of postoperative complications were comparable between the two groups (RR 1.04, 95% CI 0.91–1.18) in the meta-analysis^17^. In this study, after PSM, there were fewer minor complications in RAS (colon: 9.9% vs. 28.2%, p = 0.010; rectal: 12.5% vs. 28.1%, p = 0.214). For RAS with ERAS program, the rate was lower (colon: 6.0% vs 20.0%, p = 0.074; rectum: 11.1% vs 33.3%, p = 0.102) to conventional LSC but without significant due sample size (colon: 50; rectum: 27). Recent systemic review study for ERAS in colorectal surgery^21^, both open and LSC showed lower overall complication rate [morbidity risk ratio: 0.63 (open); 0.59 (LSC)]. There was a lower incidence of surgical site infection and anastomosis leakage. However, the risk of acute kidney injury due to restrictive hypovolemic policy was concerned in recent study^22^. The adherence rate of ERAS program was 80% in this study and the result was comparable to the conclusion of POWER study^23^ that ERAS program and microinvasive surgery can reduce LHS and the complication rate. Robotic systems are considered advanced microinvasive instruments that can reduce the complication rate. Robotic surgery with ERAS program brings significant better surgical outcome. The major complication rate for robotic ERAS after PSM was lower in colon (2.0% vs. 6.0%, p = 0.617) and similar in rectal surgeries (7.4% vs. 7.4%, p = 1.000) to conventional LSC. The leakage rate was acceptable (colon: 4.0% vs. 2.0%, p = 1.000; rectal: 7.4% vs. 0.0%, p = 0.491). The percentages of CRM positivity (7.4% vs. 7.4%, p = 1.000) and distal margin involvement (0.0% vs. 7.0%, p = 0.491) were not different. Endo-wrist and cameral control enabled the surgeon to ascertain the distal margin in the pelvis area and were all free (0/67).

The differences in candidates between the RAS and LSC groups were the confounding factors. Robotic surgery is not covered by the health insurance and is much more expensive than laparoscopic surgery in the study site. Difference in social economy status and related characteristics cannot completely be diminished after PSM. Pain scores and sexual and urological evaluations were not conducted. Cost-effectiveness was also not included in this study. The sample size was not adequate after focused on ERAS robotic assisted surgery to gain significant different in complication rate.

## Conclusions

Learning curve for robotic assisted colorectal surgery takes 31 cases. Robotic surgery with ERAS program brings significant faster recovery and fewer complication rate compared to laparoscopy in colorectal surgery.

## Supplementary Information

Supplementary Information 1.

Supplementary Information 2.

Supplementary Information 3.

Supplementary Tables.

## Data Availability

The datasets generated and analyzed during the current study are available in supplementary data file: LSC raw data de-identified, RAL raw data de-identified. The files include hospital medical record, operation note, operation nursing note, and pathology report. Most of the data in “Raw data” excel file can get from these documents. The videos dataset analyzed for detailed surgical procedure time are not uploaded due inadequate repository space for all video files but are available from the corresponding author. All data generated or analyzed during this study are included in this published article. The original data will be provided on reasonable request to corresponding author.
