# Supplementary material for: Learning curve and surgical outcome of robotic assisted colorectal surgery with ERAS program
Source: Sci Rep. 2022 Nov 29;12:20566. doi: 10.1038/s41598-022-24665-w (PMC9709162; doi:10.1038/s41598-022-24665-w)
Supplement: Supplementary file 1 — Supplementary Information 1. [file 41598_2022_24665_MOESM1_ESM.zip › LSC Raw data-Ñ[▒K/037/AR time.pdf]

AR: 12:15-14:00

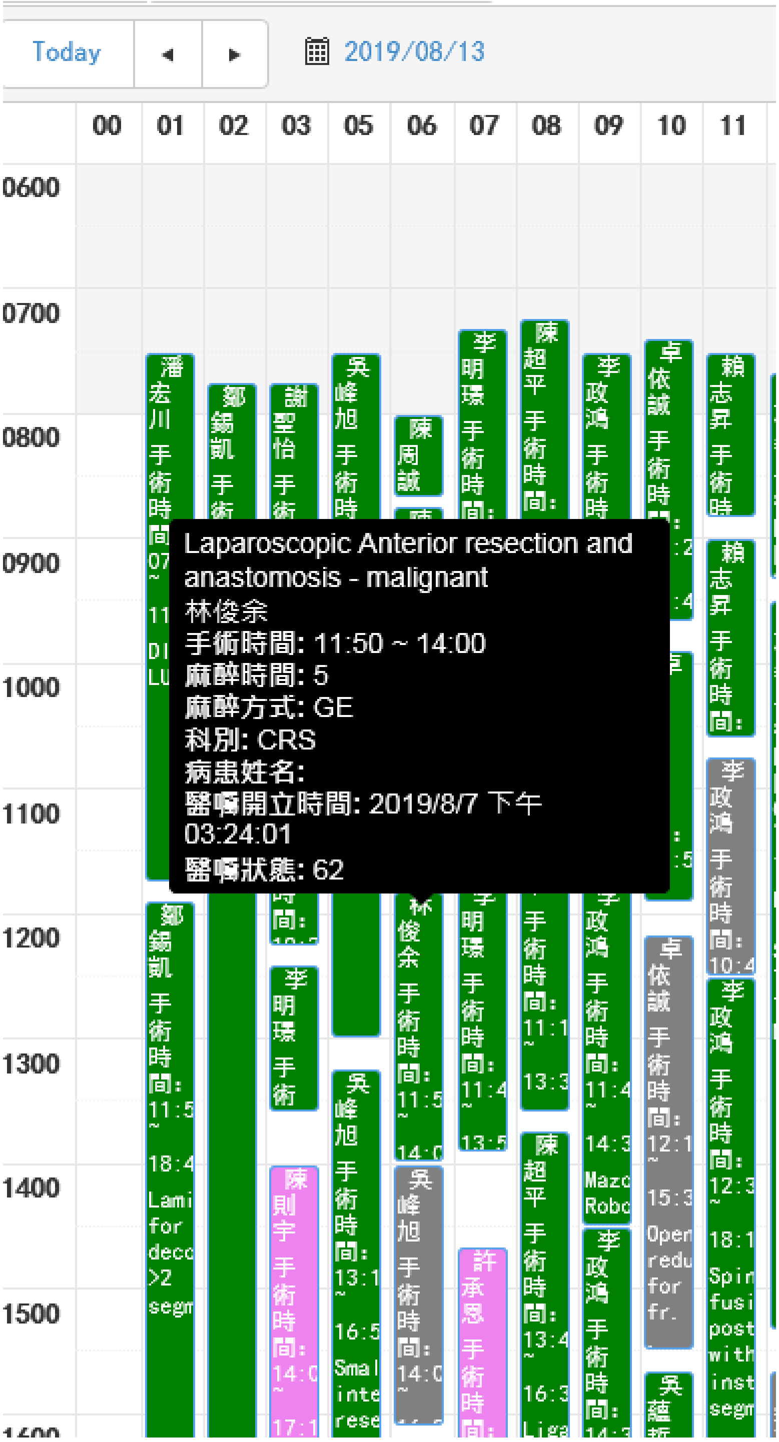
Today
2019/08/13
00 01 02 03 05 06 07 08 09 10 11
0600
0700
0800
0900
1000
1100
1200
1300
1400
1500
Laparoscopic Anterior resection and anastomosis - malignant
林俊余
手術時間: 11:50 ~ 14:00
麻醉時間: 5
麻醉方式: GE
科別: CRS
病患姓名:
醫囑開立時間: 2019/8/7 下午 03:24:01
醫囑狀態: 62
