# Supplementary figures and images for: Learning curve and surgical outcome of robotic assisted colorectal surgery with ERAS program
Source: Sci Rep. 2022 Nov 29;12:20566. doi: 10.1038/s41598-022-24665-w (PMC9709162; doi:10.1038/s41598-022-24665-w)

LAR+ hernia 1435—1925

LAR estimate 1435- 1730

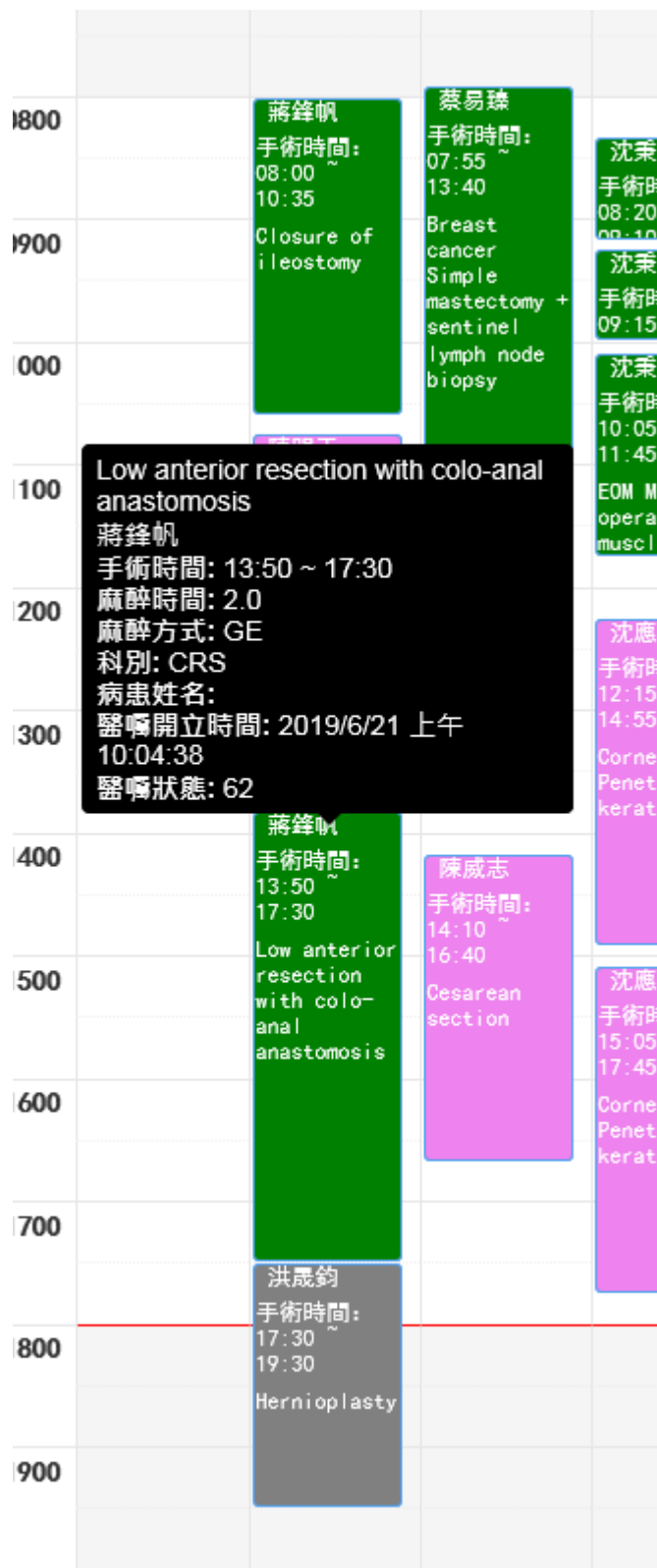

Supplement: Supplementary file 1 — Supplementary Information 1. [file 41598_2022_24665_MOESM1_ESM.zip › LSC Raw data-Ñ[▒K/028/LAR time.pdf]

RH estimated 2000-2205

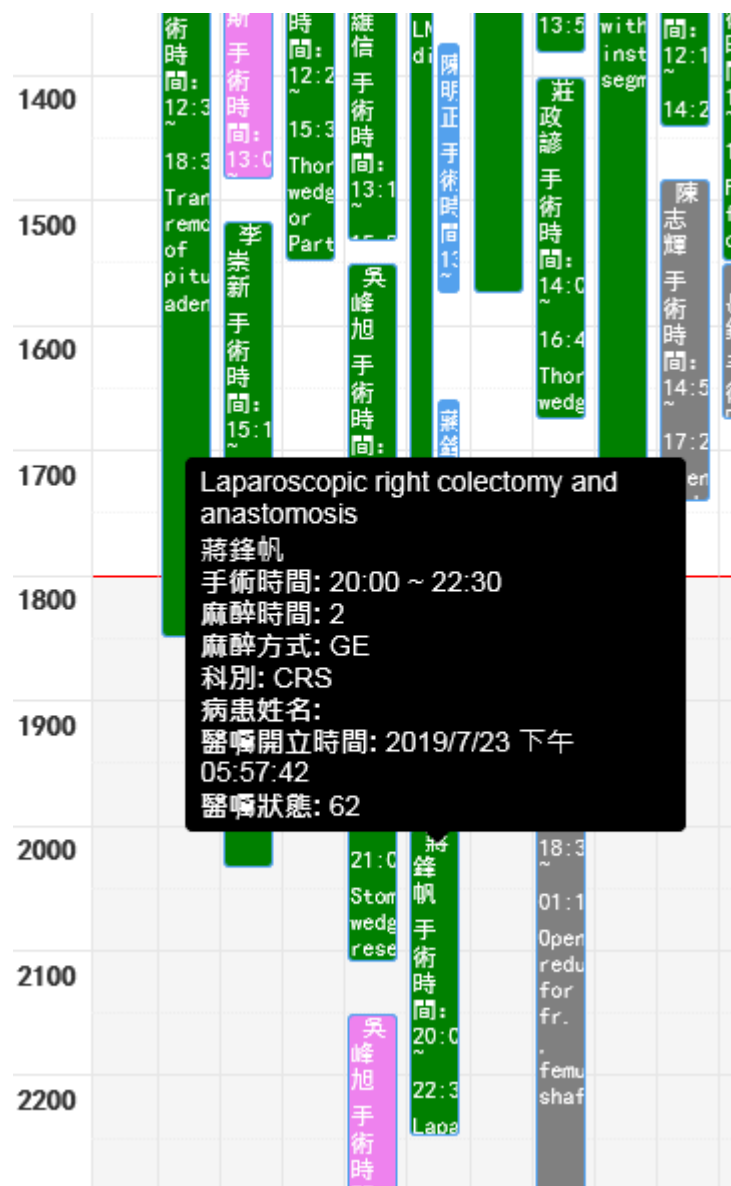

Supplement: Supplementary file 1 — Supplementary Information 1. [file 41598_2022_24665_MOESM1_ESM.zip › LSC Raw data-Ñ[▒K/032/RH time.pdf]

LAR+ URSL 1100-1615

Estimate LAR 1310-1555

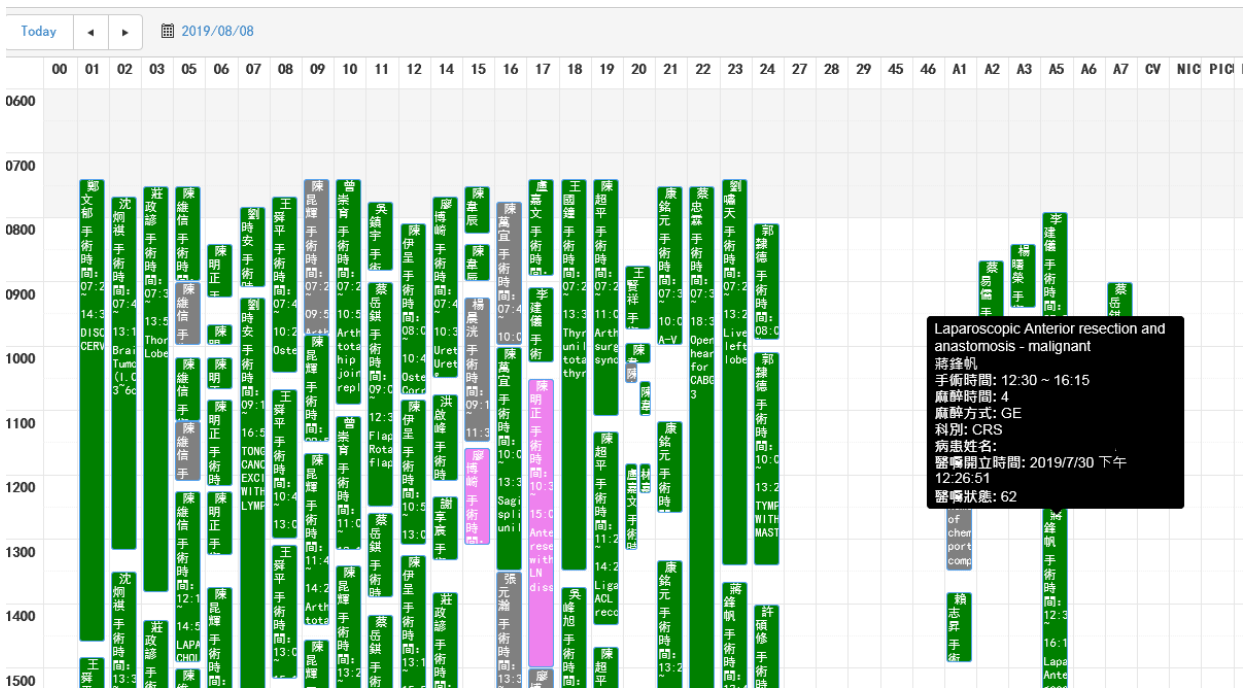

Supplement: Supplementary file 1 — Supplementary Information 1. [file 41598_2022_24665_MOESM1_ESM.zip › LSC Raw data-Ñ[▒K/035/LAR time.pdf]

Estimated AR finish time: 15:25

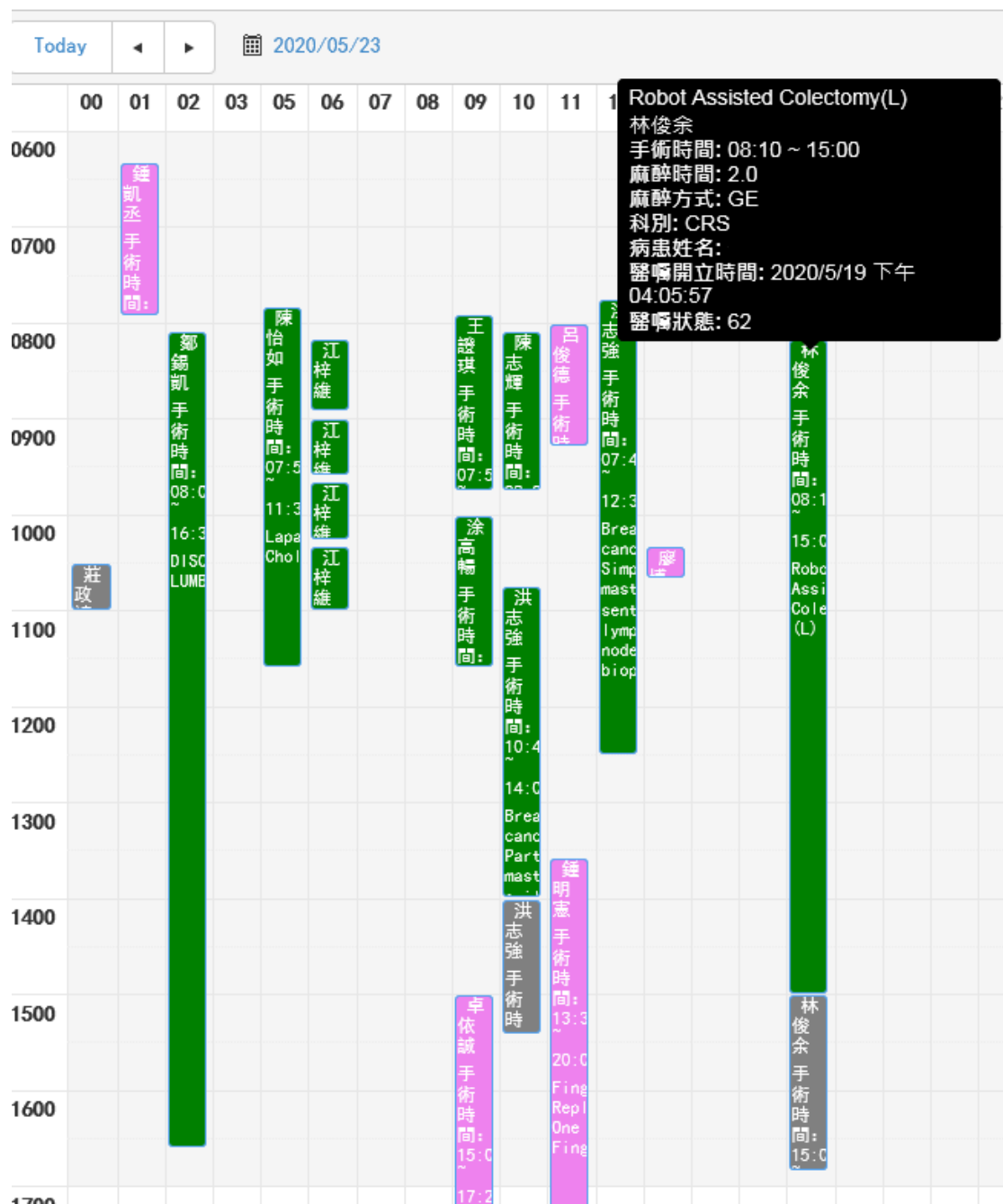

Supplement: Supplementary file 2 — Supplementary Information 2. [file 41598_2022_24665_MOESM2_ESM.zip › RAL Raw data-Ñ[▒K/007/time.pdf]

RH+ OC finish 20:20

RH estimate finish 18:50

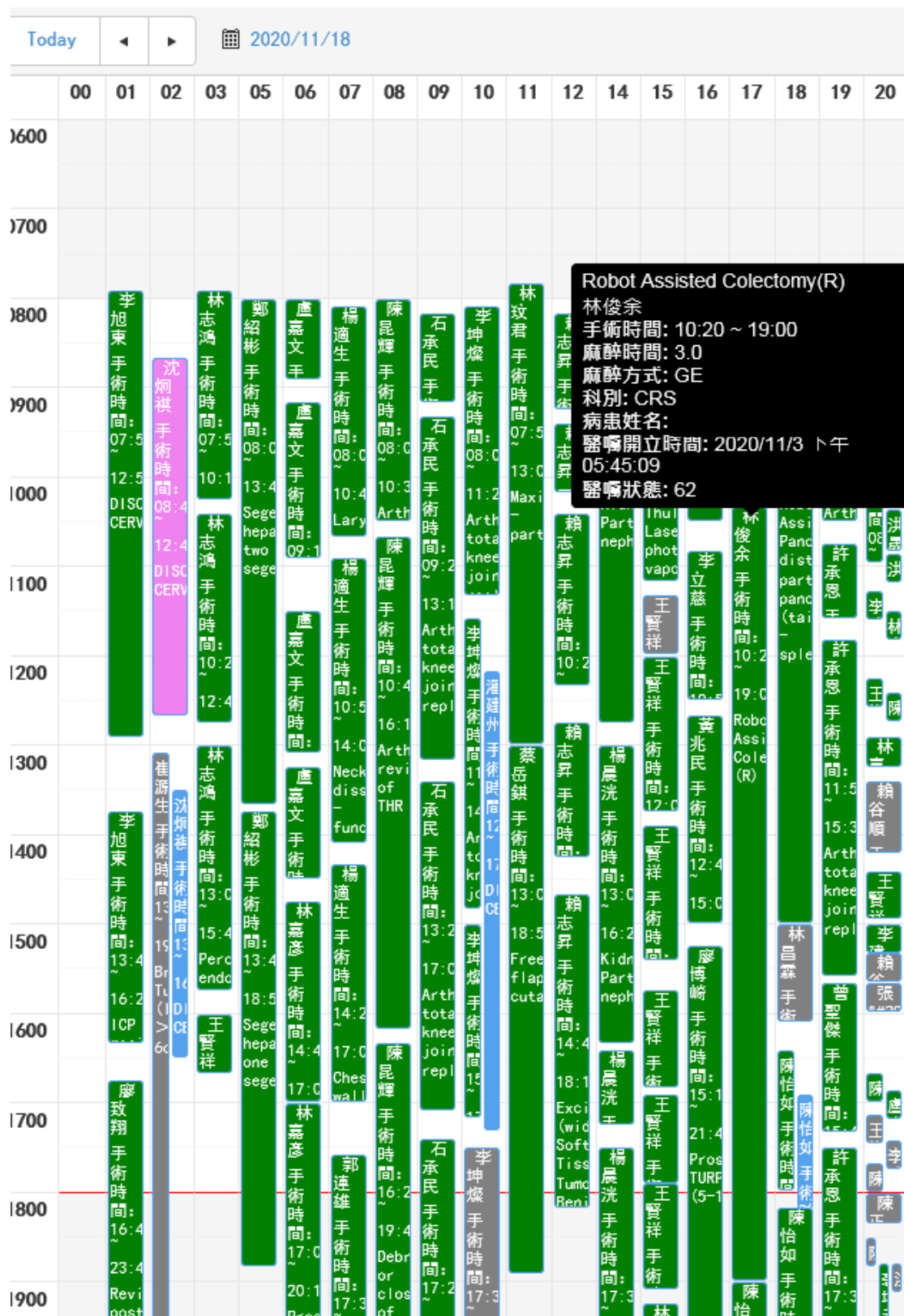

Supplement: Supplementary file 2 — Supplementary Information 2. [file 41598_2022_24665_MOESM2_ESM.zip › RAL Raw data-Ñ[▒K/032/RH time.pdf]

AR+ para-aorta LN dissection=13:35-22:30

AR finish time 20:00

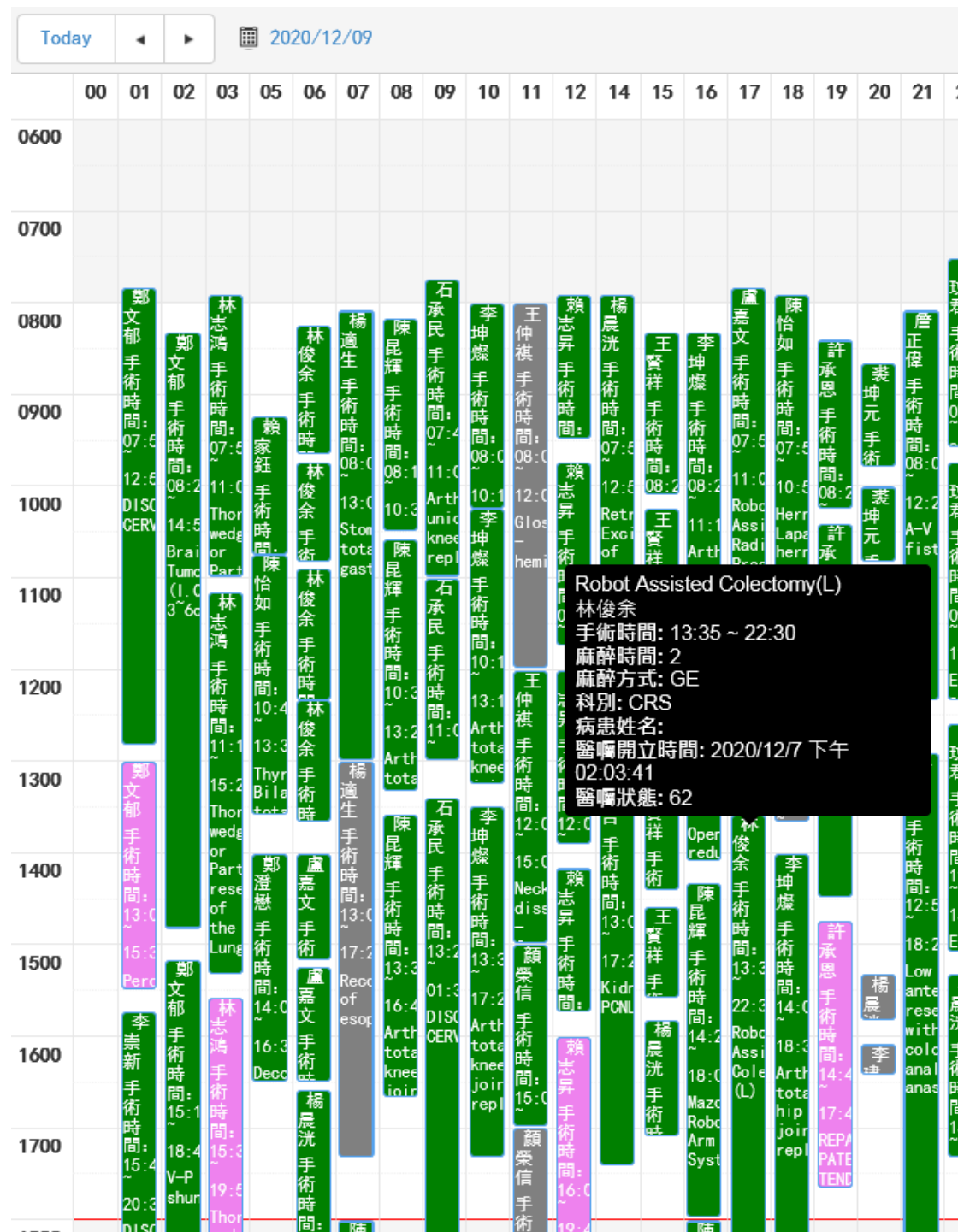

Supplement: Supplementary file 2 — Supplementary Information 2. [file 41598_2022_24665_MOESM2_ESM.zip › RAL Raw data-Ñ[▒K/042/op time.pdf]
