# Supplementary material for: Learning curve and surgical outcome of robotic assisted colorectal surgery with ERAS program
Source: Sci Rep. 2022 Nov 29;12:20566. doi: 10.1038/s41598-022-24665-w (PMC9709162; doi:10.1038/s41598-022-24665-w)
Supplement: Supplementary file 2 — Supplementary Information 2. [file 41598_2022_24665_MOESM2_ESM.zip › RAL Raw data-Ñ[▒K/004/OP time.pdf]

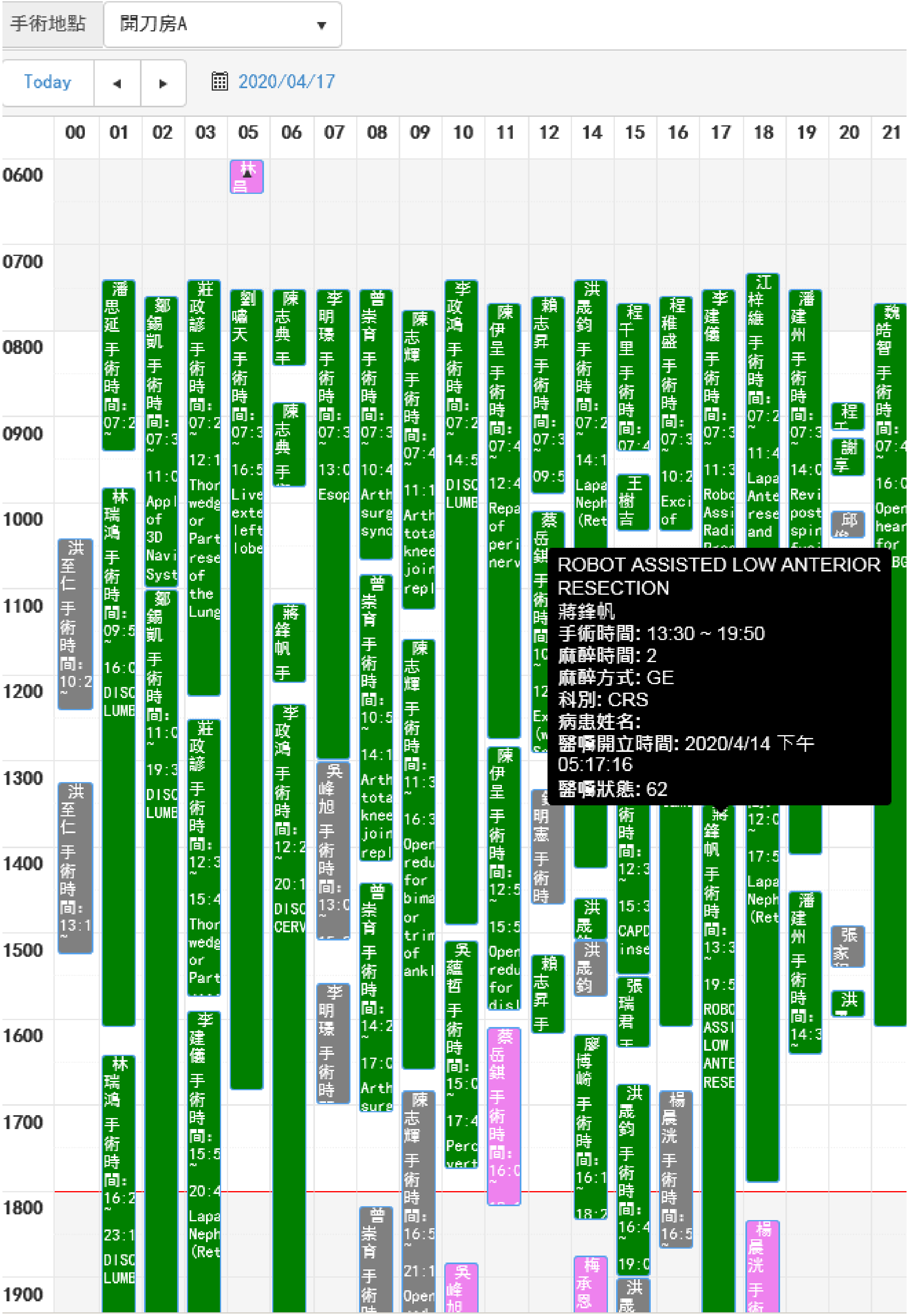
手術地點
開刀房A
Today
2020/04/17
00 01 02 03 05 06 07 08 09 10 11 12 14 15 16 17 18 19 20 21
0600
0700
0800
0900
1000
1100
1200
1300
1400
1500
1600
1700
1800
1900
ROBOT ASSISTED LOW ANTERIOR RESECTION
蔣鋒帆
手術時間: 13:30 ~ 19:50
麻醉時間: 2
麻醉方式: GE
科別: CRS
病患姓名:
醫囑開立時間: 2020/4/14 下午 05:17:16
醫囑狀態: 62
